# Association Between Trapezius Muscle Stiffness and Headache Severity in Patients With Tension‐Type Headache

**DOI:** 10.1111/ene.70393

**Published:** 2025-10-27

**Authors:** Daisuke Shimada, Eiichi Ishikawa, Takuya Kawai, Yoichi Harada, Toru Hatayama, Takuji Kono, Koichi Okamura, Toshitsugu Terakado, Kiyotaka Toyoda, Shohei Iijima, Yuta Sasaki, Satoru Miyawaki

**Affiliations:** ^1^ Department of Neurosurgery Koyama Memorial Hospital Ibaraki Japan; ^2^ Department of Neurosurgery, Institute of Medicine University of Tsukuba Ibaraki Japan; ^3^ Department of Neurosurgery Mito Brain Heart Center Ibaraki Japan; ^4^ Department of Neurosurgery Iijima Clinic Ibaraki Japan; ^5^ Department of Neurosurgery, Faculty of Medicine The University of Tokyo Tokyo Japan

**Keywords:** headache severity (HIT–6, MIDAS), muscle stiffness, risk factors and symptoms, tension‐type headache (TTH), trapezius Muscle/physiology

## Abstract

**Background:**

Tension‐type headache (TTH) is one of the most prevalent headache disorders worldwide, yet its underlying pathophysiological mechanisms remain poorly understood. Previous research suggests a potential link between myofascial factors, such as trapezius muscle stiffness, and headache severity. This study investigates the association between upper trapezius muscle stiffness and headache severity in patients with TTH.

**Methods:**

We conducted a cross‐sectional analysis of 203 patients diagnosed with TTH, evaluating muscle stiffness using a portable muscle hardness meter and headache severity using the Headache Impact Test‐6 (HIT‐6) and Migraine Disability Assessment Scale (MIDAS). Logistic regression analysis was performed to identify correlations between muscle stiffness and headache severity, adjusting for age, sex, and other clinical variables.

**Results:**

The mean muscle stiffness values were 32.5 ± 5.8 on the left and 31.3 ± 5.6 on the right. HIT‐6 scores positively correlated with muscle stiffness (*β* = 0.148), chronic TTH (*β* = 0.304), and age (*β* = −0.203). Significant clinical symptoms associated with headache severity included weakness (*β* = 0.154) and a heavy eyelid sensation (*β* = 0.154). However, shoulder stiffness and neck pain were not significantly associated with headache severity. Triggers such as stress and weather changes were also identified as significant predictors of headache severity.

**Conclusions:**

Increased trapezius muscle stiffness positively correlates with greater headache severity in TTH patients. Weakness and heavy eyelid sensation may serve as important clinical indicators of headache severity, offering potential insights for improved diagnosis and management.

## Graphical abstract

This study examined 203 patients with tension‐type headache (TTH) and found that increased trapezius muscle stiffness is associated with greater headache severity. Muscle stiffness was measured using a portable device, and headache severity was assessed using the HIT‐6 and MIDAS scales. Higher HIT‐6 scores were linked to chronic TTH, greater muscle stiffness, and younger age. Additionally, clinical symptoms like weakness and heavy eyelid sensation, as well as predictors such as weather changes and stress, were associated with more severe headaches. The findings suggest that increased trapezius muscle stiffness is associated with greater headache severity in patients with TTH.

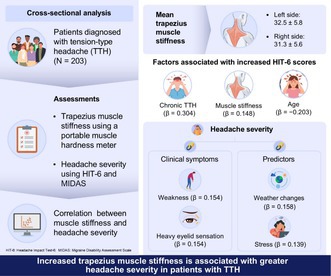

**Abbreviations**
BMI: Body mass index
HIT‐6: Headache Impact Test‐6
ICHD‐3*β*
: International Classification of Headache Disorders, 3rd edition, beta version
MIDAS: Migraine Disability Assessment Scale
TTH: Tension‐type headache

## 1 Introduction

Tension‐type headache (TTH) is the most common headache disorder, affecting an estimated 1.9 billion people worldwide, as reported by the GBD 2016 study. Among 328 diseases assessed, TTH ranks as the third most prevalent condition globally, indicating that one in four individuals experiences TTH [1]. In Japan, the prevalence rate has been reported as 22.3% [2]. Despite its high prevalence, TTH patients are less likely to seek medical care, leading to limited literature on its clinical characteristics. One proposed pathophysiological mechanism of TTH involves myofascial dysfunction [3]. Previous studies have shown that individuals with neck and shoulder pain exhibit increased trapezius muscle stiffness [4, 5]. Research using pressure‐based devices has demonstrated that muscle stiffness values are effective for assessing shoulder stiffness [6]. However, the relationship between trapezius muscle stiffness and headache severity in TTH patients remains unclear. This study hypothesizes that increased upper trapezius stiffness is associated with greater headache severity in TTH patients. The objective of this study is to elucidate the relationship between muscle stiffness values and headache severity in patients with TTH.

## 2 Patients and Methods

This study included consecutive patients diagnosed with TTH at two specialized headache clinics between July 2022 and November 2024. The diagnosis of TTH was based on the International Classification of Headache Disorders, 3rd edition (ICHD‐3*β*) [6]. Patients were included if they had undergone cervical X‐rays and muscle stiffness measurements using a portable muscle hardness meter during their initial visit. Patients with incomplete muscle stiffness measurements, those with orthopedic conditions, individuals over 75 years of age, and those who had undergone acupuncture or rehabilitation within 1 week before their visit were excluded.

**FIGURE 1:**
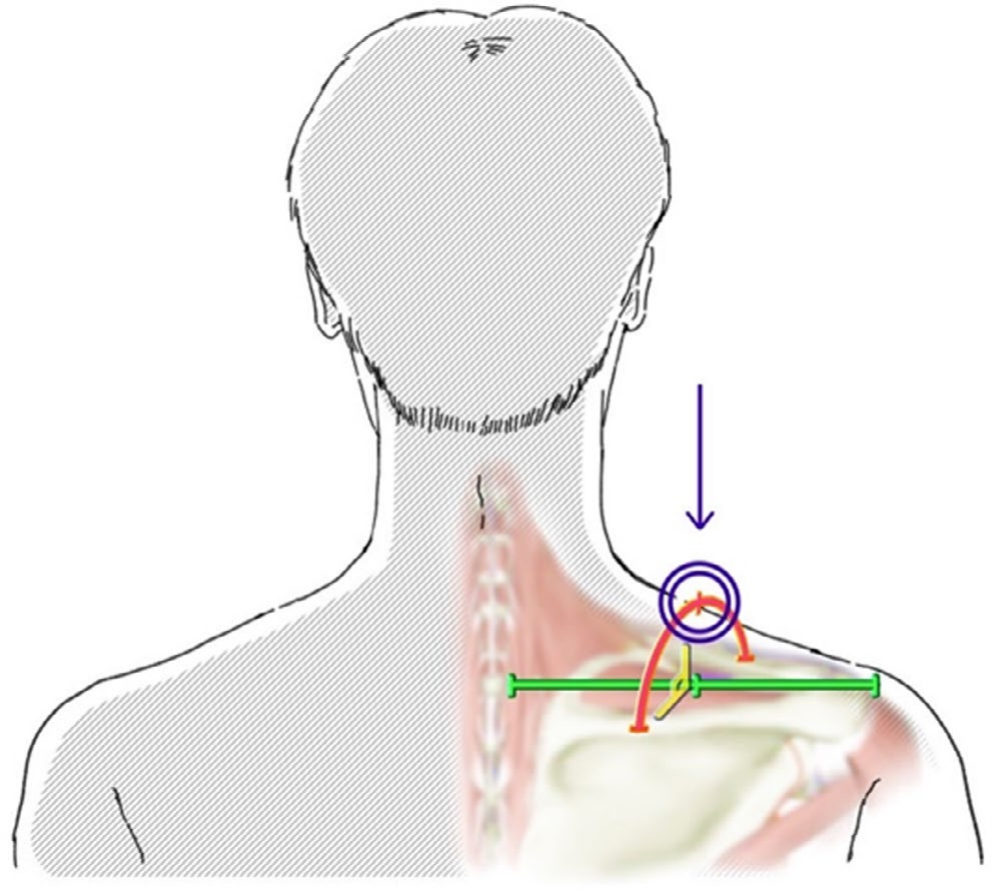
Assessment site of the upper trapezius muscle. The assessment site of the upper trapezius muscle was defined as the midpoint between the C7 spinous process and the acromion, intersecting with the midpoint between the clavicle and scapula. Muscle stiffness measurements were conducted using a portable muscle hardness meter (NEUTONE TDM‐N1), with participants seated in a standardized posture to ensure measurement consistency and accuracy.

Muscle stiffness was quantitatively assessed using a portable muscle hardness meter (NEUTONE TDM‐N1, TRY‐ALL Co. Ltd., Chiba, Japan). Participants were seated in a height‐adjustable chair with a backrest, maintaining a 90‐degree angle at the knees and hips, with feet flat on the floor. Their gaze was directed horizontally, and their hands were placed on their thighs in a relaxed position. The upper trapezius muscle was assessed at the midpoint between the C7 spinous process and the acromion, intersecting with the midpoint between the clavicle and scapula [7]. Three measurements were taken on each side, and the mean value was recorded as the muscle stiffness value. The higher value from either side was recorded as the maximum muscle stiffness (Figure 1).

Headache severity was assessed using the Headache Impact Test (HIT‐6) and the Migraine Disability Assessment Scale (MIDAS). According to ICHD‐3*β*, TTH was classified into infrequent episodic TTH (ieTTH), frequent episodic TTH (feTTH), and chronic TTH (cTTH). For analysis, the infrequent and frequent subtypes were grouped as episodic TTH (eTTH) and compared with chronic TTH (cTTH). Associations between headache severity and various clinical symptoms were also examined.

Categorical variables were analyzed using chi‐square tests or Fisher's exact tests, while logistic regression was applied for continuous variables. Odds ratios (ORs) and 95% confidence intervals (CIs) were calculated, with statistical significance set at *p* < 0.05. Multiple logistic regression analysis was performed to identify potential predictive factors, adjusting for age and sex. Bidirectional interactions were examined using logistic regression. All statistical analyses were conducted using SPSS software version 26 (SPSS, Chicago, IL, USA). This study was approved by the ethics committees of Koyama Memorial Hospital and Mito Brain Heart Center.

## 3 Results

**FIGURE 2:**
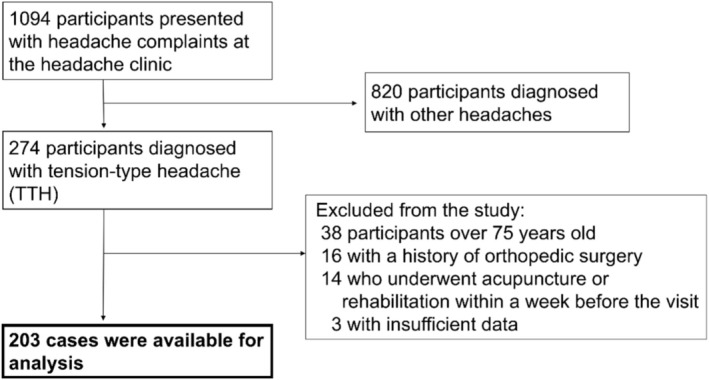
Flowchart of the cohort examining the association between trapezius stiffness and HIT‐6 scores in tension‐type headache.

A total of 203 patients were included in the study (Figure 2), with a mean age of 43 years (range: 9–75 years), and 57% (115 cases) were female. The mean height was 163 cm, the mean weight was 60.8 kg, and the mean BMI was 22.8 kg/m^2^. Trapezius muscle stiffness values were 32.5 tone on the left side and 31.3 tone on the right side. The distribution of maximum trapezius muscle stiffness values among five age categories (< 20, 20–34, 35–50, 50–64, and ≥ 65 years) showed no statistically significant differences (Kruskal–Wallis test, *p* = 0.233), suggesting that trapezius stiffness does not vary substantially with age (Figure S1). The mean HIT‐6 and MIDAS scores at the initial visit were 56.3 and 6.1, respectively. Cases of cTTH accounted for 57% (117 cases), and first‐time visits represented 53% (108 cases) (Table 1).

**TABLE 1:** Baseline characteristics of patients with tension‐type headache (TTH).

	All (*N* = 203)	eTTH (*N* = 117)	cTTH (*N* = 86)
Age (years)	43 ± 18 (9–75)	43 ± 18 (9–75)	41 ± 18 (12–75)
Sex (female), *n* (%)	115 (57%)	63 (54%)	52 (55%)
Body height (cm)	163 ± 8.7	162 ± 8.9	164 ± 8.8
Body weight (kg)	60.8 ± 13.4	60.4 ± 13.0	62.4 ± 14.9
BMI (kg/m^2^)	22.8 ± 4.1	22.8 ± 3.9	23.1 ± 4.3
Trapezius stiffness (L, tone)	32.5 ± 5.8	32.2 ± 6.1	32.2 ± 5.8
Trapezius stiffness (R, tone)	31.3 ± 5.6	31.3 ± 5.3	31.0 ± 6.3
HIT‐6 score	56.3 ± 7.6	53.9 ± 8.0	59.5 ± 6.6
MIDAS score	6.1 ± 18.1	2.6 ± 5.0	10.5 ± 25.5
Clinical symptoms, *n* (%)			
Heaviness in the head	64 (32%)	30 (25%)	34 (40%)
Shoulder stiffness	113 (56%)	64 (55%)	49 (57%)
Neck pain	74 (39%)	39 (32%)	35 (41%)
Weakness	45 (22%)	21 (18%)	24 (27%)
Numbness	17 (7%)	10 (9%)	7 (8.1%)
Dizziness	34 (17%)	15 (13%)	19 (22%)
Tinnitus	28 (14%)	12 (10%)	16 (19%)
Eyelid edema (heavy eyelid sensation)	21 (10%)	5 (4%)	16 (19%)
Nasal discharge	18 (9%)	8 (7%)	10 (12%)
Conjunctival injection	14 (7%)	3 (3%)	11 (13%)
Lacrimation	12 (6%)	7 (6%)	5 (6%)
Photophobia	2 (1.0%)	2 (1.7%)	0 (0%)
Hyperacusis	15 (7.4%)	5 (4.3%)	10 (12%)
Osmophobia	7 (3.4%)	1 (0.9%)	6 (7.0%)
External triggers, *n* (%)			
Stress	77 (38%)	36 (31%)	41 (48%)
Weather changes	39 (19%)	15 (13%)	24 (28%)
Outside heat exposure	9 (4.4%)	3 (2.6%)	6 (7.0%)
Smoking	10 (4.9%)	5 (4.3%)	5 (5.8%)
Alcohol consumption	8 (3.9%)	5 (4.3%)	3 (3.5%)
Internal triggers, *n* (%)			
Fatigue	58 (29%)	32 (27%)	26 (30%)
Sleep deprivation	55 (27%)	29 (25%)	26 (30%)
Menstruation	26 (13%)	11 (9.4%)	15 (7.4%)
Oversleeping	6 (3.0%)	2 (1.7%)	4 (4.7%)

Abbreviations: BMI, body mass index; CI, confidence interval; eTTH, episodic tension type headache; cTTH, chronic tension type headache; HIT‐6, Headache Impact Test‐6; MIDAS, Migraine Disability Assessment Scale.

Mean HIT‐6 scores varied depending on clinical symptoms and external/internal triggers. Among clinical symptoms (excluding hyperacusis and osmophobia, etc., with 15 patients or less), eyelid edema (heavy eyelid sensation) (60.4, *N* = 21) had the highest score, followed by heaviness in the head (59.3, *N* = 64) and weakness (59.0, *N* = 45). Dizziness (58.9, *N* = 34) and tinnitus (58.7, *N* = 28) were also associated with elevated mean HIT‐6 scores, whereas neck pain (57.3, *N* = 74) and shoulder stiffness (56.9, *N* = 113) had slightly lower scores. Among external and internal triggers, oversleeping (63.0, *N* = 6) had the most significant impact, followed by weather changes (58.7, *N* = 39) and menstruation (58.6, *N* = 26). Stress (57.9, *N* = 77) and sleep deprivation (57.0, *N* = 55) also contributed to higher HIT‐6 scores, highlighting their role in headache severity (Table 1, Table S1).

Risk factors associated with mean HIT‐6 scores were identified using single regression analysis, revealing significant associations with age, muscle stiffness, cervical lordosis angle, MIDAS score, and cTTH. Multiple regression analysis demonstrated significant associations with muscle stiffness (*β* = 0.148, *p* = 0.024), cTTH (*β* = 0.304, *p* < 0.001), and age (*β* = −0.203, *p* = 0.002) (Table 2, Figure S1). The MIDAS score (*β* = 0.117, *p* = 0.009, single regression analysis) was also associated with the mean HIT‐6 score but was not included in the Table analysis due to strong multicollinearity. A positive correlation was found between headache severity and muscle stiffness, indicating that greater muscle stiffness was associated with increased headache severity.

**TABLE 2:** Single and multiple regression analyses of risk factors for higher HIT‐6 scores in tension‐type headache.

Risk factor	Single regression analysis			Multiple regression analysis		
	*β*	SE	*p*	*β*	SE	*p*
Age (years old)	−0.230	0.029	0.001*	−0.203	0.027	0.002*
Sex (female)	−0.070	1.077	0.322	—	—	—
Muscle stiffness (tone)	0.164	0.096	0.019*	0.148	0.089	0.024*
Body height (cm)	0.057	6.145	0.422	—	—	—
Body weight (kg)	0.026	0.040	0.709	—	—	—
BMI (kg/m^2^)	−0.001	0.133	0.986	—	—	—
Chronic TTH (yes)	0.336	1.020	< 0.001*	0.304	0.998	< 0.001*

*Note:* Values are expressed as *β* coefficients with standard errors (SEs) and *p* values.

Abbreviations: BMI, body mass index; TTH, tension‐type headache.

* Statistically significant differences (*p* < 0.05) are marked with an asterisk.

Among clinical symptoms, dizziness, weakness, and a heavy eyelid sensation were significantly associated with headache severity. Multiple regression analysis identified weakness (*β* = 0.154, *p* = 0.028) and a heavy eyelid sensation (*β* = 0.154, *p* = 0.027) as significant factors (Table 3). No significant associations were found with shoulder stiffness, neck pain, numbness, conjunctival injection, lacrimation, or tinnitus (Table 3).

**TABLE 3:** Single and multiple regression analyses of clinical symptoms for HIT‐6 scores in TTH.

Clinical symptoms	Single regression analysis			Multiple regression analysis		
	*β*	SE	*p*	*β*	SE	*p*
Heaviness in the head	0.096	1.106	0.171	—	—	—
Shoulder stiffness	−0.070	1.077	0.322	—	—	—
Neck pain	0.164	0.096	0.019*	0.156	1.114	0.424
Weakness	0.185	1.266	0.008*	0.154	1.270	0.028*
Dizziness	0.148	1.417	0.035*	0.098	1.423	0.162
Tinnitus	0.119	1.526	0.102	—	—	—
Eyelid edema (heavy eyelid sensation)	0.186	1.727	0.009*	0.154	1.725	0.027*
Nasal discharge	−0.016	1.882	0.816	—	—	—
Numbness	−0.025	2.045	0.721	—	—	—
Conjunctival injection	0.008	2.111	0.908	—	—	—
Lacrimation	0.038	2.267	0.589	—	—	—
Photophobia	−0.011	5.429	0.877	—	—	—
Hyperacusis	0.109	2.033	0.122	—	—	—
Osmophobia	0.120	2.918	0.089	—	—	—

*Note:* Values are expressed as *β* coefficients with SEs and *p* values. Multivariate logistic regression was used to determine statistical significance.

* Statistically significant differences (*p* < 0.05) are marked with an asterisk.

External and internal triggers significantly associated with headache severity included physical or emotional stress, weather changes, and oversleeping. In contrast, no associations were found with alcohol consumption, smoking, outside heat exposure, or fatigue (Table 4). Multiple regression analysis identified weather changes (*β* = 0.158, *p* = 0.022) and stress (*β* = 0.139, *p* = 0.044) as the strongest predictors of headache severity (Table 4).

**TABLE 4:** Single and multiple regression analyses of triggers for HIT‐6 scores in TTH.

Triggers	Single regression analysis			Multiple regression analysis		
	*β*	SE	*p*	*β*	SE	*p*
Stress	0.163	1.092	0.021*	0.139	1.075	0.044*
Weather changes	0.150	1.346	0.033*	0.158	1.321	0.022*
Outside heat	0.123	2.585	0.081	—	—	—
Smoking	0.011	2.478	0.876	—	—	—
Alcohol consumption	−0.032	2.755	0.649	—	—	—
Fatigue	0.070	1.185	0.321	—	—	—
Sleep deprivation	0.054	1.206	0.447	—	—	—
Menstruation	0.045	1.934	0.521	—	—	—
Oversleeping	0.153	3.129	0.029*	0.121	3.151	0.088
Bathing	0.106	3.836	0.133	—	—	—

*Note:* Values are expressed as *β* coefficients with SEs and *p* values. Multivariate logistic regression was used to determine statistical significance.

* Statistically significant differences (*p* < 0.05) are marked with an asterisk.

When analyzing the association between mean HIT‐6 scores and headache subtype (eTTH vs. cTTH), the cTTH group had significantly higher HIT‐6 scores than the eTTH group (59.5 vs. 53.9), indicating a greater impact on daily functioning among patients with cTTH (Table 1). In the eTTH group, only the symptom of heaviness in the head was significantly associated with mean HIT‐6 scores (*β* = 0.272, *p* = 0.003), and no internal or external triggers were identified as significant contributors. In contrast, the cTTH group exhibited broader symptomatology: both weakness and tinnitus were associated with higher HIT‐6 scores, with weakness remaining a significant independent predictor in multivariate analysis (*β* = 0.241, *p* = 0.026). Additionally, among various triggers, stress (*β* = 0.299, *p* = 0.003) and exposure to external heat (*β* = 0.232, *p* = 0.024) were significantly associated with higher HIT‐6 scores exclusively in the cTTH group. Although scatter plots suggested slightly elevated trapezius muscle stiffness and mean HIT‐6 scores in cTTH, no statistically significant differences in stiffness values were observed between the groups (left: 32.2 vs. 32.2; right: 31.3 vs. 31.0; *p* = 0.605). Moreover, analysis for all TTH and subgroup analysis for eTTH demonstrated a significant positive correlation (Figure S2AB). In contrast, no significant correlation was observed in cTTH (Figure S2C). These findings suggest that trapezius muscle stiffness may reflect headache impact in eTTH but not in cTTH patients. These findings suggest that cTTH is characterized by greater headache severity, a wider range of associated symptoms, and more distinct triggering factors compared to eTTH (Tables S2–5, Figure S2).

## 4 Discussion

The present study revealed that among TTH cases, higher headache severity was associated with cTTH and greater maximum muscle stiffness. The potential pathophysiological mechanisms of TTH include genetic factors, myofascial mechanisms (such as myofascial nociception), and mechanisms of chronicity (including central sensitization and altered descending pain regulation). Previous studies on myofascial mechanisms have reported increased muscle tenderness and stiffness in TTH cases compared to healthy individuals [8, 9]. Additionally, beyond increased tenderness, pericranial muscles have been found to be stiffer in TTH cases [10, 11]. Muscle stiffness can be quantitatively assessed using a muscle hardness meter, which allows for quick and convenient measurement within approximately 1 min in a clinical setting [7]. The trapezius muscle, evaluated in this study, is one of the largest superficial skeletal muscles and has recently received increased attention for its fascial involvement in primary headache disorders. The upper trapezius muscle is easily accessible for manual examination, and palpation of fascial trigger points within this muscle has been reported to induce headache attacks [12]. The trapezius muscle is innervated by the anterior branch of the cervicospinal nerve, linking it to the trigeminocervical complex [13]. The association between cervical pathology and headache may be attributed to the convergence of nociceptive input from the upper cervical spine and the trigeminal system at the level of the trigeminal nucleus [14]. This convergence mechanism explains the ability to reproduce headaches by applying pressure to the cervical structures, thereby sensitizing the innervated areas of the trigeminocervical nucleus [15]. This sensitization can be quantified using algometry and has been shown to exhibit reduced values in the craniocervical region. However, it should be noted that muscle stiffness tends to be higher when muscles are fatigued and lower when they are relaxed, as seen after acupuncture and rehabilitation [16]. Other studies have concluded that patients with TTH have lower neck muscle thickness and lower tenderness thresholds than healthy controls [17]. Additionally, MRI studies have shown that T2 values of the trapezius muscle are significantly associated with both the number of headache days and the presence of neck pain. Higher T2 values correlate with a greater number of headache days and are expected to serve as a potential biomarker for headache severity [18]. Increased sensitivity of the pericranial musculature to pressure is observed in both eTTH and cTTH patients. However, cTTH patients exhibit greater sensitivity than eTTH patients, and this muscle sensitivity varies with pressure [19, 20]. Pressure sensitivity has been suggested to increase in proportion to the severity and frequency of TTH episodes [21].

Currently, no biomarker exists to validate the association between headache frequency and neck pain using an objective, measurable correlation beyond self‐report or manual surveys [22]. Muscle hardness meters are simple, easy to use in clinical settings, and may serve as an objective indicator of headache severity.

Additionally, this study found that weakness (*β* = 0.154, *p* = 0.028) and a heavy eyelid sensation (*β* = 0.154, *p* = 0.027) were associated with headache severity as presenting symptoms at the initial visit. No significant association was found with subjective shoulder stiffness or perceived neck pain, which is consistent with previous studies indicating that trapezius stiffness does not directly reflect subjective shoulder stiffness [7]. To our knowledge, this is the first study to report that patients presenting with both weakness and ocular symptoms alongside headache experience greater headache severity. Further research is needed to validate these findings and investigate underlying mechanisms.

This study has several limitations. First, muscle stiffness values may vary depending on the measurement location, as the position of the trapezius muscle differs among individuals, leading to variations in evaluation sites. In particular, individuals with a hunched posture often exhibit forward‐protruding shoulder blades, which may result in stiffness measurements capturing the scapular region rather than the intended site. To ensure consistency and reliability, we strictly standardized the evaluation site across all participants. Second, the study population consisted exclusively of Japanese participants, preventing us from assessing potential ethnic differences. Third, for the initial 42 participants, no evaluation was conducted to determine whether massage or acupuncture had been applied before the examination, introducing the possibility of underestimated stiffness values. Fourth, we were unable to analyze ieTTH and feTTH separately, which may have influenced the interpretation of findings related to eTTH. Fifth, a contemporaneous healthy control group was not included during the study period. However, to provide a reference point, we conducted preliminary measurements of trapezius muscle stiffness in 20 headache‐free healthy volunteers (mean age 42.2 years; 50% female), with average muscle stiffness values of 21.9 (right side) and 22.0 (left side), and a mean height and weight of 1.64 m and 60.0 kg, respectively. In addition, we did not evaluate muscular strength or bulk, which may influence muscle stiffness values. However, we assessed body height, weight, and BMI, and found no significant differences across groups. Furthermore, measurements were taken at the initial visit, when many patients were likely experiencing an acute headache, although some may have had milder or intermittent symptoms. If patients had undergone massage or similar interventions just before the initial visit, stiffness values may have been underestimated. Moreover, we did not perform longitudinal follow‐up measurements of muscle stiffness across acute and recovery phases and therefore could not demonstrate temporal changes related to headache improvement. Furthermore, while 78.8% of patients reported the use of acute analgesics and no patients were on prophylactic therapy, the potential influence of medication use cannot be entirely excluded; however, it is likely to have been minimal in this cohort. These factors should be considered as additional limitations of our study. Future prospective studies should include age‐ and sex‐matched control groups to enable more rigorous comparisons. Lastly, the cross‐sectional design of this study did not allow for the assessment of longitudinal changes in trapezius muscle stiffness following treatment. Consequently, we were unable to evaluate whether stiffness values decreased in parallel with clinical improvement in individual patients. Nonetheless, previous research has indicated that although headache severity, duration, and chronicity did not directly correlate with muscle stiffness in TTH patients, those who showed clinical improvement exhibited a reduction in muscle stiffness over time during follow‐up [10].

In conclusion, this study found that TTH patients with more severe headaches exhibited higher maximum muscle stiffness. Additionally, patients presenting with weakness and ocular symptoms experienced greater headache severity, which may have clinical implications.

## Author Contributions

Study concept and design: **D. S**, **E. I**., data collection: **D. S**, **T. K**., **Y. H**., **T. H**., **T. K**., **K. O**., **T. T**., **K. T**., **S. I**., **Y. S**., Analysis and interpretation of data: **D. S**, **E. I**., Drafting of the manuscript: **D. S**, Revising of the manuscript: **E. I**., **S. M**., Supervising of the study: **E. I**.

## Conflicts of Interest

The authors declare no conflicts of interest.

## Supporting information


**Data S1:** Supporting Information.

## Data Availability

The data that support the findings of this study are available from the corresponding author upon reasonable request.

## References

[1] V. L. Feigin , A. A. Abajobir , K. H. Abate , et al., “Global, Regional, and National Burden of Neurological Disorders During 1990–2015: a Systematic Analysis for the Global Burden of Disease Study 2015,” Lancet Neurology 16, no. 11 (2017): 877–897. 28931491 10.1016/S1474-4422(17)30299-5 PMC5641502

[2] F. Sakai and H. Igarashi , “Prevalence of Migraine in Japan: a Nationwide Survey,” Cephalalgia 17, no. 1 (1997): 15–22. 10.1046/j.1468-2982.1997.1701015.x 9051330

[3] S. Ashina , D. D. Mitsikostas , M. J. Lee , et al., “Tension‐Type Headache,” Nature Reviews Disease Primers 7, no. 1 (2021): 24. 10.1038/s41572-021-00257-2 33767185

[4] E. Cerezo‐Tellez , M. Torres‐Lacomba , O. Mayoral‐Del Moral , B. Sanchez‐Sanchez , J. Dommerholt , and C. Gutierrez‐Ortega , “Prevalence of Myofascial Pain Syndrome in Chronic Non‐Specific Neck Pain: A Population‐Based Cross‐Sectional Descriptive Study,” Pain Medicine 17, no. 12 (2016): 2369–2377. 28025371 10.1093/pm/pnw114

[5] H. T. Leong , F. Hug , and S. N. Fu , “Increased Upper Trapezius Muscle Stiffness in Overhead Athletes With Rotator Cuff Tendinopathy,” PLoS One 11, no. 5 (2016): 155187. 10.1371/journal.pone.0155187 PMC4861275 27159276

[6] Headache Classification Committee of the International Headache Society (IHS) the International Classification of Headache Disorders, vol. 38, 3rd ed. (Cephalalgia, 2018), 1–211. 10.1177/0333102417738202 29368949

[7] T. Sawada , H. Okawara , D. Nakashima , et al., “Reliability of Trapezius Muscle Hardness Measurement: A Comparison Between Portable Muscle Hardness Meter and Ultrasound Strain Elastography,” Sensors (Basel) 20, no. 24 (2020): 7200. 33339151 10.3390/s20247200 PMC7765603

[8] M. Ashina , L. Bendtsen , R. Jensen , F. Sakai , and J. Olesen , “Muscle Hardness in Patients With Chronic Tension‐Type Headache: Relation to Actual Headache State,” Pain 79, no. 2–3 (1999): 201–205. 10068165 10.1016/s0304-3959(98)00167-5

[9] L. Bendtsen , S. Ashina , A. Moore , and T. J. Steiner , “Muscles and Their Role in Episodic Tension‐Type Headache: Implications for Treatment,” European Journal of Pain 20, no. 2 (2016): 166–175. 26147739 10.1002/ejp.748

[10] F. Sakai , S. Ebihara , M. Akiyama , and M. Horikawa , “Pericranial Muscle Hardness in Tension‐Type Headache. A Non‐Invasive Measurement Method and Its Clinical Application,” Brain 118, no. Pt 2 (1995): 523–531. 7735892 10.1093/brain/118.2.523

[11] M. A. Huysmans , B. M. Blatter , and A. J. van der Beek , “Perceived Muscular Tension Predicts Future Neck‐Shoulder and Arm‐Wrist‐Hand Symptoms,” Occupational and Environmental Medicine 69, no. 4 (2012): 261–267. 22213837 10.1136/oemed-2011-100279

[12] M. N. Landgraf , J. T. Biebl , T. Langhagen , et al., “Children With Migraine: Provocation of Headache via Pressure to Myofascial Trigger Points in the Trapezius Muscle? ‐ A Prospective Controlled Observational Study,” European Journal of Pain 22, no. 2 (2018): 385–392. 28952174 10.1002/ejp.1127

[13] S. Ashina , D. D. Mitsikostas , M. J. Lee , et al., “Tension‐Type Headache,” Nature Reviews. Disease Primers 7, no. 1 (2021): 24. 10.1038/s41572-021-00257-2 33767185

[14] D. H. Watson and P. D. Drummond , “The Role of the Trigemino Cervical Complex in Chronic Whiplash Associated Headache: A Cross Sectional Study,” Headache 56, no. 6 (2016): 961–975. 27091393 10.1111/head.12805

[15] D. H. Watson and P. D. Drummond , “Cervical Referral of Head Pain in Migraineurs: Effects on the Nociceptive Blink Reflex,” Headache 54, no. 6 (2014): 1035–1045. 24666216 10.1111/head.12336

[16] M. Murayama , K. Nosaka , T. Yoneda , and K. Minamitani , “Changes in Hardness of the Human Elbow Flexor Muscles After Eccentric Exercise,” European Journal of Applied Physiology 82, no. 5–6 (2000): 361–367. 10985588 10.1007/s004210000242

[17] J. Muniz , A. G. Flor , D. D. Balmaseda , D. M. Vera , A. S. Sierra , and G. G. P. Sevilla , “Pain Sensitization and Atrophy of Deep Cervical Muscles in Patients With Chronic Tension‐Type Headache,” Revista da Associação Médica Brasileira (1992) 69, no. 10 (2023): e20230841. 37729231 10.1590/1806-9282.20230841 PMC10511287

[18] N. Sollmann , P. Schandelmaier , D. Weidlich , et al., “Headache Frequency and Neck Pain Are Associated With Trapezius Muscle T2 in Tension‐Type Headache Among Young Adults,” Journal of Headache and Pain 24, no. 1 (2023): 84. 37438700 10.1186/s10194-023-01626-w PMC10337094

[19] L. Bendtsen , R. Jensen , and J. Olesen , “Decreased Pain Detection and Tolerance Thresholds in Chronic Tension‐Type Headache,” Archives of Neurology 53, no. 4 (1996): 373–376. 8929161 10.1001/archneur.1996.00550040113021

[20] J. Schoenen , D. Bottin , F. Hardy , and P. Gerard , “Cephalic and Extracephalic Pressure Pain Thresholds in Chronic Tension‐Type Headache,” Pain 47, no. 2 (1991): 145–149. 1762808 10.1016/0304-3959(91)90198-7

[21] V. Ulrich , M. Gervil , and J. Olesen , “The Relative Influence of Environment and Genes in Episodic Tension‐Type Headache,” Neurology 62, no. 11 (2004): 2065–2069. 15184615 10.1212/01.wnl.0000129498.50793.8a

[22] Z. Liang , L. Thomas , G. Jull , and J. Treleaven , “The Neck Disability Index Reflects Allodynia and Headache Disability but Not Cervical Musculoskeletal Dysfunction in Migraine,” Physical Therapy 102, no. 5 (2022): 27. 10.1093/ptj/pzac027 PMC9156011 35230421

