# Supplementary material for: Association Between Trapezius Muscle Stiffness and Headache Severity in Patients With Tension‐Type Headache
Source: Eur J Neurol. 2025 Oct 27;32(10):e70393. doi: 10.1111/ene.70393 (PMC12554949; doi:10.1111/ene.70393)
Supplement: Supplementary file 1 — Data S1: Supporting Information. [file ENE-32-e70393-s001.docx]

**Supplementary Table 1**

**Relationship between HIT-6 scores and clinical symptoms, external triggers, and internal triggers in patients with tension-type headache (TTH)**

| **Variable** | **HIT-6 Score (Mean ± SD)** | |
| --- | --- | --- |
|  | **With** | **Without** |
| **Clinical symptoms** |  |  |
| Heaviness in the head | 59.3±5.7 | 55.0±8.0 |
| Shoulder stiffness | 56.9±7.2 | 55.8±7.9 |
| Neck pain | 57.3±6.7 | 55.8±8.1 |
| Weakness | 59.0±6.7 | 55.6±7.7 |
| Numbness | 55.7±7.8 | 56.4±7.6 |
| Tinnitus | 58.7±5.8 | 56.0±7.8 |
| Eyelid edema | 60.4±4.9 | 55.9±7.7 |
| Nasal discharge | 55.9±6.2 | 56.4±7.7 |
| Conjunctival injection | 56.6±7.6 | 56.3±7.6 |
| Lacrimation | 57.5±6.6 | 56.3±7.7 |
| Photophobia | 57.7±6.7 | 56.3±7.6 |
| Hyperacusis | 59.3±7.1 | 56.1±7.6 |
| Osmophobia | 61.1±5.0 | 56.2±7.6 |
| **External triggers** |  |  |
| Stress | 57.9±7.4 | 55.4±7.6 |
| Weather changes | 58.7±5.7 | 55.8±7.9 |
| Outside heat | 60.7±4.3 | 56.1±7.7 |
| Smoking | 56.7±7.6 | 56.3±7.6 |
| Alcohol consumption | 55.1±9.2 | 56.4±7.6 |
| **Internal triggers** |  |  |
| Fatigue | 57.2±7.4 | 56.0±7.7 |
| Sleep deprivation | 57.0±7.5 | 56.1±7.7 |
| Menstruation | 58.6±7.2 | 56.0±7.6 |
| Oversleeping | 63.0±5.4 | 56.1±7.6 |

The mean and standard deviation (SD) of HIT-6 scores are shown for patients with and without each clinical symptom, external trigger, and internal trigger in tension-type headache (TTH).

**Supplementary Table 2: Single regression analysis of clinical symptoms for HIT-6 in episodic TTH**

| **Clinical symptoms** | **β** | **SE** | ***p-*value** |
| --- | --- | --- | --- |
| Heaviness in the head | 0.272 | 1.648 | 0.003* |
| Shoulder stiffness | 0.077 | 1.668 | 0.410 |
| Neck pain | 0.102 | 1.771 | 0.271 |
| Weakness | 0.085 | 2.163 | 0.360 |
| Tinnitus | 0.038 | 2.779 | 0.690 |
| Eyelid edema | 0.034 | 4.110 | 0.711 |
| Nasal discharge | -0.080 | 3.286 | 0.386 |
| Numbness | -0.039 | 2.975 | 0.672 |
| Conjunctival injection | -0.070 | 5.248 | 0.451 |
| Lacrimation | 0.051 | 3.504 | 0.582 |
| Photophobia | -0.014 | 6.413 | 0.882 |
| Hyperacusis | -0.002 | 3.654 | 0.984 |
| Osmophobia | -0.017 | 9.027 | 0.851 |

Values are expressed as β coefficients with standard errors (SEs) and p values. No statistically significant differences are observed for any of the symptoms except heaviness in the head.

**Supplementary Table 3: Single regression analysis of external and internal triggers for HIT-6 in episodic TTH**

| **Triggers** | **β** | **SE** | ***p*-value** |
| --- | --- | --- | --- |
| Stress | -0.013 | 1.806 | 0.886 |
| Weather changes | 0.050 | 2.488 | 0.593 |
| Outside heat | 0.002 | 5.261 | 0.983 |
| Smoking | -0.073 | 4.102 | 0.429 |
| Alcohol consumption | -0.106 | 4.089 | 0.252 |
| Fatigue | 0.056 | 1.867 | 0.548 |
| Sleep deprivation | -0.028 | 1.929 | 0.763 |
| Menstruation | 0.029 | 3.296 | 0.759 |
| Oversleeping | -0.028 | 1.929 | 0.763 |
| Bathing | 0.309 | 9.029 | 0.973 |

Values are expressed as β coefficients with SEs and p values. No statistically significant differences are observed for any of the triggers.

**Supplementary Table 4**

**Single and multiple regression analyses of clinical symptoms for HIT-6 in chronic TTH**

| **Clinical symptoms** | **Single regression analysis** | | | **Multiple regression analysis** | | |
| --- | --- | --- | --- | --- | --- | --- |
|  | **β** | **SE** | ***p*-value** | **β** | **SE** | ***p*-value** |
| Heaviness in the head | 0.096 | 1.106 | 0.171 | — | — | — |
| Shoulder stiffness | 0.064 | 1.355 | 0.540 | — | — | — |
| Neck pain | 0.017 | 1.367 | 0.872 | — | — | — |
| Weakness | 0.251 | 1.470 | 0.014* | 0.241 | 1.460 | 0.026* |
| Tinnitus | 0.209 | 1.728 | 0.042* | 0.098 | 1.643 | 0.361 |
| Eyelid edema | 0.034 | 4.110 | 0.711 | — | — | — |
| Nasal discharge | -0.031 | 2.206 | 0.768 | — | — | — |
| Numbness | 0.039 | 3.030 | 0.707 | — | — | — |
| Conjunctival injection | -0.092 | 2.107 | 0.374 | — | — | — |
| Lacrimation | 0.035 | 2.591 | 0.739 | — | — | — |
| Photophobia | 0.019 | 2.956 | 0.862 | — | — | — |
| Hyperacusis | 0.154 | 2.133 | 0.158 | — | — | — |
| Osmophobia | -0.017 | 9.027 | 0.851 | — | — | — |

Values are expressed as β coefficients with SEs and p values. Statistically significant differences (p < 0.05) are marked with an asterisk (*). Multivariate logistic regression was used to determine statistical significance.

**Supplementary Table 5**

**Single regression analysis of external and internal triggers for HIT-6 in chronic TTH**

| **Triggers** | **β** | **SE** | ***p*-value** |
| --- | --- | --- | --- |
| Stress | 0.299 | 1.293 | 0.003* |
| Weather changes | 0.161 | 1.499 | 0.120 |
| Outside heat | 0.232 | 2.521 | 0.024 |
| Smoking | 0.062 | 3.027 | 0.551 |
| Alcohol consumption | 0.079 | 3.860 | 0.448 |
| Fatigue | 0.056 | 1.867 | 0.548 |
| Sleep deprivation | 0.175 | 1.462 | 0.090 |
| Menstruation | 0.059 | 2.203 | 0.573 |
| Oversleeping | 0.137 | 3.340 | 0.186 |
| Bathing | 0.309 | 9.029 | 0.973 |

Values are expressed as β coefficients with SEs and p values. No statistically significant differences are observed for any of the triggers except stress-related ones.

**Supplementary Figure 1. Distribution of trapezius muscle stiffness across different age groups in patients with tension-type headache**


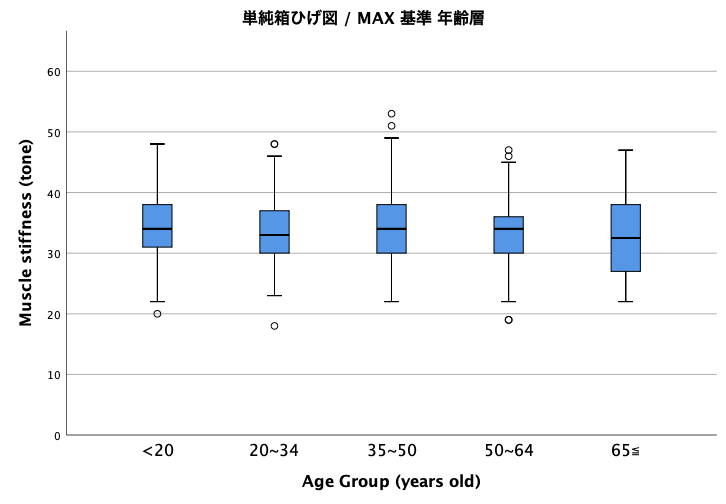


Box-and-whisker plots represent the distribution of maximum trapezius muscle stiffness (in tone units) across five age groups: <20, 20–34, 35–50, 50–64, and ≥65 years. Each box indicates the interquartile range (IQR), with the horizontal line representing the median. Whiskers extend to 1.5 times the IQR, and circles represent outliers. No statistically significant differences were observed among the age groups (Kruskal–Wallis test, p = 0.233), suggesting that trapezius stiffness does not significantly vary with age.

**Supplementary Figure 2. Association Between Muscle Stiffness and HIT-6 Scores in Patients with Episodic and Chronic TTH**

**
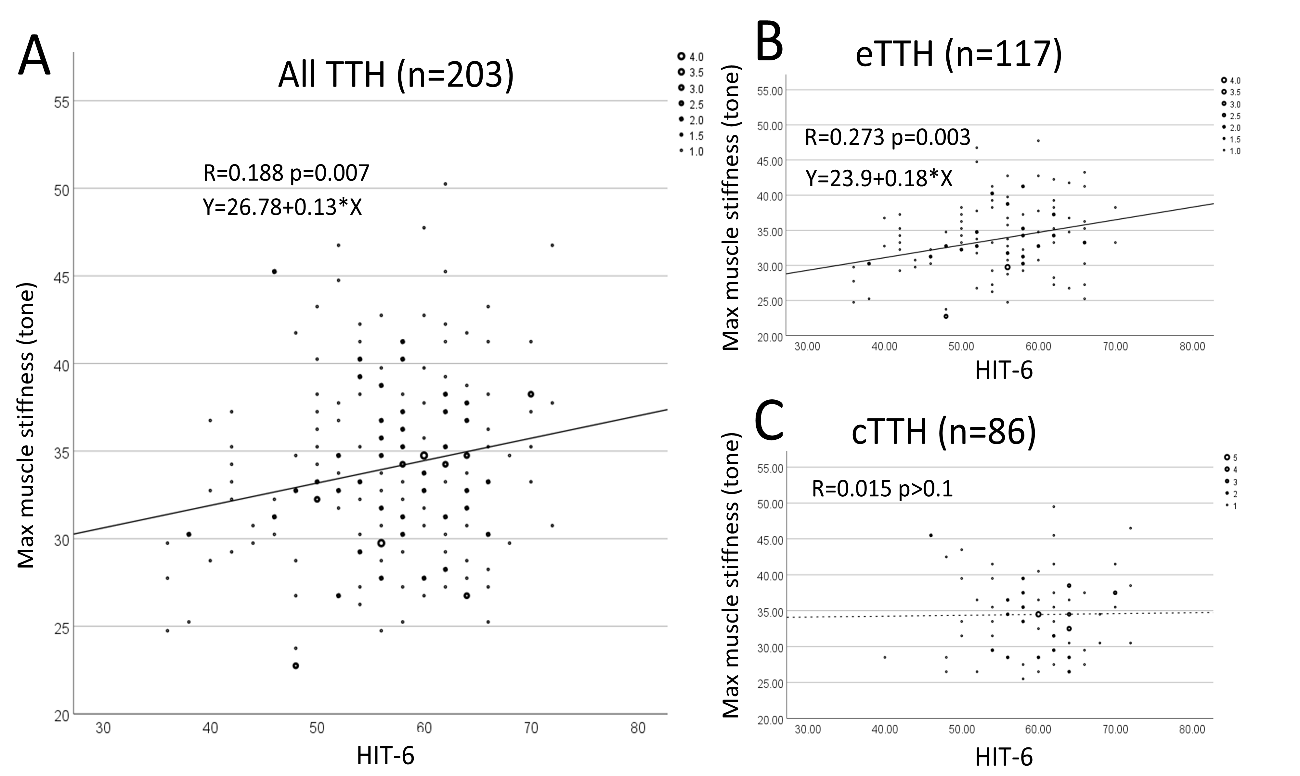
**

Scatter plot illustrating the correlation between baseline HIT-6 scores and trapezius muscle stiffness in patients with all tension-type headache (all TTH; A), episodic tension-type headache (eTTH; B) and chronic tension-type headache (cTTH; C). Each dot represents an individual patient, and dot size indicates the frequency of overlapping data points.
